# Alternative Splicing in Human Viral Oncogenesis and Tumor Progression

**DOI:** 10.3390/cancers18122004

**Published:** 2026-06-20

**Authors:** Ilaria Martelli, Lucia Annamaria Cappabianca, Paola Cipriani, Antonietta Rosella Farina, Maddalena Sbaffone, Andrew Reay Mackay

**Affiliations:** Department of Biotechnological and Applied Clinical Sciences, University of L’Aquila, Via Vetoio, 67100 L’Aquila, Italy; ilaria.martelli@graduate.univaq.it (I.M.); luciaannamaria.cappabianca@univaq.it (L.A.C.); paola.cipriani@univaq.it (P.C.); maddalena.sbaffone@univaq.it (M.S.); andrewreay.mackay@univaq.it (A.R.M.)

**Keywords:** group 1 carcinogenic viruses, host-cell alternative splicing, viral activation of host-cell oncogenes, viral inactivation of host-cell tumor suppressors, hallmarks of cancer

## Abstract

Oncogenic viruses are responsible for between 12% and 20% of human cancers worldwide. They trigger tumorigenesis by integrating into host-cell genomes, altering cell cycle pathways, and evading immune detection. Oncoviral cancers exhibit low rates of mutation, implicating alternative splicing as an underappreciated alternative mechanism for oncogene and oncogenic pathway activation in oncoviral pathogenesis and progression. In order to create alternatively spliced viral proteins for replication and viral genome maintenance, oncoviruses take advantage of host-cell splicing machinery. Some of these proteins inhibit major host-cell tumor suppressors to promote the proliferation of DNA-damaged host-cells in order to facilitate persistent infection, whilst others interact with and de-regulate the expression and activity of host-cell splicing factors to alter host transcript splice site selection. The latter reprograms host-cell transcriptomes to express aberrant, sometimes oncogenic protein isoforms, which interact with oncoviral proteins to promote host-cell transformation and subsequent tumor progression to metastatic disease. In this article, we review oncovirus-induced alternative splicing as a fundamental, underappreciated, oncogenic and tumor-promoting mechanism. We present detailed descriptions of individual human oncoviruses. We compare how these oncoviruses hijack host-cell splicing mechanisms, how specific aberrant alternatively spliced host-cell protein isoforms, induced by oncoviruses, influence tumor pathogenesis and progression, organized with respect to the hallmarks of cancer, and provide a section on therapeutic perspectives. This approach not only crystallizes the complexity of how oncovirus-induced host-cell alternative splicing can influence cancer pathogenesis and progression but also reveals novel potential therapeutic opportunities.

## Simple Summary

Oncogenic viruses cause tumors by facilitating the proliferation of DNA-damaged infected cells and by hijacking the host-cell RNA splicing machinery, reprogramming transcriptomes to express aberrant, sometimes directly oncogenic protein isoforms that drive tumor progression from initiation to metastatic disease, and influence all of the hallmarks of cancer. Despite wide-ranging complexity, better understanding of how this underappreciated splicing-centric mechanism is regulated by different oncoviruses reveals novel potential therapeutic opportunities to correct virus-induced oncogenic splicing.

## 1. Introduction

Cancers are multifactorial diseases, in which genetic, environmental and lifestyle factors contribute to neoplastic transformation and progression. As of 2026, the International Agency for Research on Cancer (IARC) has listed nine viruses as Group 1 carcinogens in humans: human papillomavirus, hepatitis B virus, hepatitis C virus, hepatitis D virus, Epstein–Barr virus, Kaposi sarcoma-associated herpesvirus, Merkel cell polyomavirus, human T-lymphotropic virus type-1 and HIV. These viruses are considered to be responsible for between 12 and 20% of all human cancers worldwide [1,2,3,4].

Although most viral infections are resolved by interactive inflammatory and immune responses, oncogenic viruses have evolved mechanisms to evade these responses [5]. This, together with host immunosuppressive conditions; directed or accidental viral genome integration into host genomes; alternatively spliced viral oncoproteins; host-cell tumor suppressor inactivation either by viral genome insertion, direct inactivation or degradation by viral oncoproteins, and aberrant activation of host-cell oncogenes and oncogenic pathways by alternative splicing, are the main mechanisms through which Group 1 carcinogenic viruses drive infected cells towards neoplastic transformation and promote tumor progression [6,7,8].

An underestimated mechanism through which Group 1 oncoviruses promote cellular transformation, tumor initiation, tumor progression, therapeutic resistance and metastatic progression, is the corruption of host-cell splicing mechanisms, resulting in the expression of aberrant alternatively spliced protein isoforms that have potential to influence one or more of the hallmarks of cancer, from sustained proliferation, resistance to cell death, replicative immortality, angiogenesis, invasion and metastasis, immune evasion, and genomic instability, to tumor promoting inflammation [9,10,11].

Pre-mRNA splicing is a tightly regulated physiological process that plays a key role in the regulation of gene expression, and alternative splicing is the process through which a single gene generates multiple mRNA transcripts and subsequent protein isoforms, contributing to cellular complexity and functional diversity [12]. The association between alternative splicing and cancer is well established [10,11]. In cancer, furthermore, aberrant alternative splicing is now recognized to be a significantly more frequent mechanism in activating oncogenes and oncogenic pathways than genetic mutations in a wide range of different cancers, and in particular, cancers that exhibit low mutation rates, which include those induced by oncoviruses [10,13].

The most common causes of tumor-associated alternative splicing are mutations in splicing regulatory elements, de-regulated splicing factor expression and epigenetic alterations to chromatin [14]. Within this context, oncoviruses hijack the host-cell splicing machinery to facilitate the expression of functionally different alternatively spliced viral protein isoforms, from limited genomes, to optimize viral replication and minimize detection and elimination by the host’s inflammatory and immune responses. These alternatively spliced viral oncoproteins also corrupt the host-cell splicing machinery, resulting in cellular oncogene and oncogenic pathway activation and the inactivation of tumor suppressors and tumor-suppressing signaling pathways. This places oncovirus-induced host-cell alternative splicing at the forefront in improving understanding of how oncoviruses induce cellular transformation, promote tumor metastatic progression and influence response to therapy [15].

Oncovirus hijacking of host-cell splicing machinery is primarily involved in promoting the alternative splicing of viral transcripts that facilitate viral replication and evasion of host inflammatory and immune responses [16]. They do this by either directly interacting with SR and hnRNPs splicing factors, by altering splicing factor expression and/or by sequestering pre-mRNA processing factors, including splicing factors in specific stress-induced nuclear bodies [17,18], forcing cells to produce transcriptomes composed of specific, sometimes oncogenic or tumor-suppressing splice variants that not only support virus survival but induce cellular transformation, resulting in tumor initiation and promotion of tumor progression [18,19].

In this article, following a brief introduction into splicing and alternative splicing mechanisms, we present a comprehensive narrative review of how Group 1 human carcinogenic viruses hijack and corrupt alternative splicing mechanisms in equivalent ways, by altering the expression, localization, or activity of core spliceosome components and splicing regulatory factors. This subversion of host-cell alternative splicing not only allows oncogenic viruses to generate pro-oncogenic viral transcript isoforms but also to activate host-cell oncogenes and oncogenic pathways, and inhibit tumor suppressors and tumor-suppressor signaling pathways, the effects of which impact all of the hallmarks of cancer from neoplastic transformation to metastatic progression.

## 2. Alternative Splicing and Viral Oncogenesis

### 2.1. Alternative Splicing

**Figure 1:**
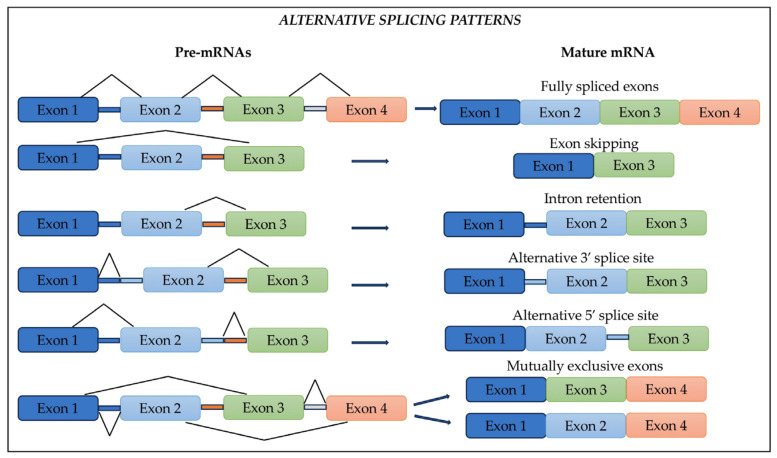
Schematic representation of the various ways that alternative splicing can alter protein isoform expression.

Splicing is a fundamental post-transcriptional mechanism that is required to eliminate intronic sequences from pre-mRNAs and facilitate the accurate joining of exon sequences that encode the protein sequence. Alternative splicing is the regulated post-transcriptional process through which exons of a single pre-mRNA are joined in different combinations to generate multiple protein isoforms from a single gene, by selecting or eliminating specific exons through exon skipping or alternative splice site use. This increases transcriptome and proteome complexity, and occurs in approximately 95% of human genes (Figure 1) [11,20].

Both constitutive and alternative splicing are catalyzed by a large molecular machine called the spliceosome, which is composed of 5 small-nuclear RNAs (U1, U2, U4, U5, and U6) and over 100 auxiliary proteins. Small nuclear RNAs are core components that associate with proteins to form nuclear ribonucleoproteins (snRNPs) [21], which bind specific splice sites and catalyze the splicing reaction. U1 snRNP binds to the donor 5′ splice site, U2 snRNP binds to the branch point sequence, and U4/U6-U5 tri-snRNP brings the snRNPs together to form the pre-catalytic spliceosome. The splicing process initiates with the recognition of specific consensus sequences in the pre-mRNA: specifically, the donor 5′ splice site, the acceptor 3′ splice site, the branch point (BPS), and the polypyrimidine tract (PPT). The spliceosome catalyzes two chemical trans-esterification reactions; in the first reaction, the intron folds into a lariat, freeing its 5′ end, followed by the second reaction that joins exon sequences together [22].

Non-snRNP components include SR proteins, rich in serine and arginine, that bind to exon splicing enhancers that facilitate snRNP recruitment, whereas hnRNPs bind frequently to splicing silencers and inhibit splicing. U2AF binds to the PPT, SF1 that binds to the BPS during the assembly stage, and the RNA helicases Brr2m, Prp2, Prp16 and Prp43, that restructure the spliceosome at different stages, resulting in spliceosome activation, facilitating the release of intron and exon products [20,22].

Subtle changes in spliceosome complexity drive alternative splicing in a condition-dependent manner, resulting in the generation of tissue, microenvironment, and cell cycle phase-specific protein isoforms [20]. Alterations in this process, induced by mutations in splice, splicing enhancer or splicing silencer sites, or by alterations in splicing factor expression and activity, can lead to aberrant splicing events that can activate oncogenes and oncogenic pathways and/or inactivate tumor suppressors and tumor-suppressing pathways [10].

### 2.2. Viral Modulation of Alternative Splicing in Host and Viral Transcripts

Viruses have evolved multiple strategies to manipulate host gene expression at the post-transcriptional level. In cancers caused by oncogenic viruses, the hijacking of host-cell splicing mechanisms is not only a critical target for the generation of different protein isoforms from viral genomes but also generates aberrant splicing of host-cell genomes. These alternatively spliced protein isoforms optimize viral replication and protect viral genomes by promoting host-cell proliferation and survival but, in doing so, also increase the potential for neoplastic transformation [23], and can be broadly classified into direct mechanisms, involving viral manipulation of the splicing machinery, and indirect mechanisms, resulting from virus-induced alterations in cellular stress responses and signaling pathways [24].

Many carcinogenic viruses directly modify the expression levels or activation of host SR splicing factors, promoting alternative splicing [25,26,27], exemplified by high-risk HPVs that enhance the expression of several SR proteins (e.g., SRSF1, SRSF2, SRSF3 and SRSF10) that influence both viral and host-cell alternative splicing, with tumor-initiating and tumor-progression potential [28]. Carcinogenic viruses also alter hnRNP expression [29,30], exemplified by EBV, which not only alters hnRNP expression but also activity, altering splice site selection, which results in aberrant alternative splicing and aberrant hnRNP isoforms that promote cell proliferation, survival, and immune evasion [31]. Oncoviruses also alter host-cell alternative splicing indirectly by modifying transcriptional dynamics, chromatin organization, nuclear stress granule formation, and by activating cellular signaling pathways that regulate splicing [17,18].

Aberrant host-cell alternative splicing events can also be induced by viral oncoproteins, exemplified by SV40 polyomaviruses’ large T antigen induction of alternative splicing of the neurotrophin receptor tropomyosin related kinase TrkA in neuroblastoma cells, which results in expression of the alternatively spliced TrkAIII oncoprotein, also detected in polyomavirus MCPyV-positive Merkel cell carcinomas, identifying a polyomavirus-dependent mechanism for host-cell oncogene and oncogene pathway activation through alternative splicing [32,33,34].

Oncogenic viruses extensively exploit host-cell splicing machinery to generate alternative splicing of transcripts for viral protein expression, exemplified by alternative splicing of early polyomavirus transcripts that generate the large T antigen oncoprotein or the multiple early (E) transcripts generated from a single polycistronic pre-mRNA that regulate the switch from viral replication to the expression of early E oncoproteins that inhibit host-cell p53 and pRb tumor suppressors function [35,36,37].

**Figure 2:**
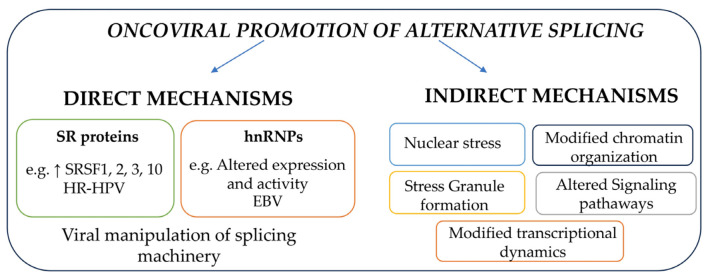
Schematic representation of the direct and indirect mechanisms through which oncogenic viruses promote alternative splicing to drive oncogenesis and tumor progression.

The promotion of alternative splicing by oncoviruses, therefore, not only represents an underestimated mechanism in oncogenesis, tumor progression, and response to therapy but also a therapeutic target (Figure 2).

## 3. Overviews of Specific Human Oncoviruses

### 3.1. Human Papillomavirus (HPV)

Human papillomavirus (HPVs), a group of non-enveloped double-stranded DNA viruses of the Papillomaviridae family, represent the most common sexually transmitted viral infection worldwide, and are recognized as causative in approximately 5% of all human cancers, including cervical, vaginal, vulvar, penile, anal, and oropharyngeal cancers [38,39]. The majority of HPV infections remain asymptomatic or sub-clinic unless the immune system is compromised and, if left untreated, become persistent, increasing the potential for transformation [40].

Based on the correlation between specific HPV subtypes and oncogenesis, HPVs are classified into high-risk and low-risk groups. HPV-16 and HPV-18 are considered high-risk, oncogenic and to cause cervical, anal, vaginal, vulvar, penile, and oropharyngeal cancers [41].

HPVs localise, replicate and persist in diverse stratified epithelial cell-types and utilize host-cell pathways to protect, replicate and maintain viral genomes by stimulating host-cell proliferation and survival, and by evading detection by the immune system [40].

#### 3.1.1. Genomic Organization and Oncoproteins (E5, E6, E7)

The HPV circular genomic episome encodes multiple proteins necessary for initiation, maintenance and completion of viral replication, the expression of which depends upon alternative splicing [42], regulated in turn by the differentiation status of host-cells, exemplified by HPV E5, E6, and E7 oncoproteins, which are essential for viral replication and subvert cellular differentiation. The balance in E6 and E7 expression is also regulated by alternative splicing from the same pre-mRNA and is critical for host-cell transformation and subsequent tumor progression [43].

#### 3.1.2. Alternative Splicing of Viral Transcripts

Various splice sites have been identified within the E6 and E7 coding regions of high-risk HPVs, which are absent in low-risk HPVs [43]. In line with their oncogenic activity, the E6 oncoprotein promotes p53 degradation and inactivation via the ubiquitination pathway [44]. In contrast, the E7 oncoprotein interacts with and inhibits retinoblastoma family pRb, p107, and p130 proteins, disrupting their interaction with transcription factors of the E2F family, permitting infected cells to override the G1/S cell cycle checkpoint, resulting in the aberrant proliferation of DNA-damaged cells [45].

By promoting the activity of E2F transcription factors, HPV oncoproteins also increase expression of the cellular splicing factor SRSF10, detected in HPV-positive cervical cancer [46], leading to SRSF10-mediated alternative splicing of several mRNAs involved in oncogenic pathway activation. These include the growth promoting oncogenic BCLAF1 alternatively spliced isoform BCLAF1-L; the membrane-associated mIL1PAR alternatively spliced isoform of IL1RAP that upregulates CD46 “don’t eat me” signalling to evade macrophage phagocytosis, and binds and stabilizes the MYB proto-oncogene increasing glycolysis and the tumor promoting macrophage M2 phenotype; the BIN1(12+) alternatively spliced isoform of BIN1 that interacts with ANXA1 to promote an inflammation suppressing TME and drug-resistance; the SREK1L alternatively spliced isoform of SREK1 that promotes proliferation, the PKM2 isoform of PK involved in the “Warburg effect” and the anti-apoptotic BCL-xL alternatively spliced isoform, representing an impressive array of alternative splicing isoforms with tumor initiating and tumor progressing potential [46,47,48,49].

The alternatively spliced E6 isoform is also subject to alternative splicing, resulting in expression of the E6*I isoform, which shares 44 amino acids with full-length E6, and is characterized by intron removal, altering the E6 open-reading frame to add an extra 13 amino acids and a novel stop codon. E6*I also exhibits oncogenic activity by impairing mitochondrial function and dysregulating the expression of genes associated with ROS metabolism [50,51]. E6*I mRNA expression distinguishes replication cycles of high-risk from low-risk HPVs [52,53,54].

The HPV16 oncovirus also expresses the alternatively spliced E5 protein, which exhibits anti-apoptotic and immune-evading activity, and synergizes with E6 and E7 to promote tumor progression [55].

#### 3.1.3. HPV Interactions with the Host-Cell Splicing Machinery

Alternative splicing is not only critical for the production of proteins from the HPV genome, but is also critical for HPV oncoprotein-induced host-cell transformation and subsequent progression of HPV-positive cancers [36]. This is achieved by hijacking and modifying the host-cell splicing machinery in order to produce the full suite of viral mRNAs. HPV alternative splicing is regulated by serine/arginine-rich (SR) proteins and cycles of SR-protein phosphorylation and dephosphorylation [56], and by antagonizing heterogeneous ribonucleoproteins (hnRNPs), which typically exercise negative control over splicing [57].

SR and hnRNP proteins contribute to HPV mRNA alternative splicing and HPV oncoproteins bind to specific splicing factors to induce aberrant alternative splicing events in HPV-positive cervical cancers. These interactions result in significant upregulation of host-cell genes, including splicing factors, that alter metabolism and promote tumorigenesis [36]. SR and hnRNP proteins are overexpressed in HPV-positive cervical pre-cancerous changes and, therefore, potentially control aberrant oncogenic alternative splicing of host gene transcripts that promote transformation and tumor genesis. [58,59]. This is supported by the detection of higher levels of SRSF1, SRSF2 and SRSF3 expression in the mid to upper layers of infected keratinocytes and in tissue samples from patients with transient HPV infection [58], and linked to the capacity of the HPV alternatively spliced viral E2 transcription factor, which binds and trans-activates SR gene promoters [60].

The HPV E2 protein also plays a crucial role in the HPV life cycle and pathogenicity, and is involved in viral genome replication, transcription and segregation [61], interacts with SR SRSF1, 2, 4, 5 and 7 proteins [62,63,64,65], and also binds other spliceosome components and cellular RNA processing factors, to further corrupt host-cell splicing machinery. E2 may also influence host-cell splicing by recruiting splicing factors to RNA, in a manner similar to the recruitment of polyadenylation factors [66]. Although E2 clearly alters host-cell transcript alternative splicing, the molecular mechanisms involved remain to be fully elucidated.

HnRNP proteins also regulate early alternative splicing events induced by HR-HPV 16. HR-HPV16 stimulates the SRPK1–SRSF axis in keratinocytes, resulting in cytoplasmic SRSF1 localization during differentiation, and associates with increased levels of both cytoplasmic and nuclear phosphorylated forms of SRSF1 [67]. The HPV alternatively spliced E4 protein, a biomarker for active HPV infection, binds SRPK1 to prevent SR SRSF1, SRSF3, SRSF4, and SRSF7 protein phosphorylation to influence pre-mRNA processing [68], resulting in aberrant splicing of host-cell transcripts [36].

### 3.2. Human Gammaherpesvirus Epstein–Barr Virus (EBV, HHV-4)

Epstein–Barr virus (EBV), also known as human herpesvirus 4 (HHV-4), belongs to the Herpesviridae family. EBV, due to ease of transmission via saliva, blood transfusions and organ transplants, is considered to be present in > 95% of the global population [69,70], and infects B-cells, epithelial cells and T-cells [71].

EBV infection in immunocompromised patients may result in lymphomas, such as Burkitt lymphoma, Hodgkin lymphoma, and B-cell lymphomas, may induce epithelial cancers, such as nasopharyngeal and gastric carcinomas, and has also been linked to non-malignant conditions, including infectious mononucleosis, oral hairy leukoplakia, systemic lupus erythematosus and multiple sclerosis [72,73].

Primary infection is usually asymptomatic but 35% to 50% of infected adolescents develop infectious mononucleosis approximately one month after exposure. Following acute infection, virus-specific T cell responses result in latent state EBV persistence [74,75,76,77]. This latent EBV infective state can be interrupted by conditions that depress the immune system, leading to EBV replication, which increases the potential for the transformation of infected lymphoid and epithelial cells [78,79,80].

#### 3.2.1. The EBV Genome and Transforming Activity

The large (172–175 kb) double-stranded EBV DNA genome encodes more than 80 ORFs and non-coding RNAs [81,82]. In vitro, EBV drives human B-lymphocyte transforming proliferation, resulting in lymphoblastoid cell lines (LCLs), highlighting the B-cell lymphotropic nature of EBV. Most EBV-infected LCLs do not produce active EBV virions but express EBV genome-derived alternatively spliced EBNA 1, 2, 3A, 3B, 3C and LP six nuclear antigens, LMP 1, 2A and 2B membrane proteins, EBER1, EBER2, a snoRNA non-coding RNAs and 44 miRNAs, which are essential for promoting and sustaining host-cell proliferation and survival [83].

#### 3.2.2. EBV Regulation of Alternative Splicing

Several EBV proteins drive or modulate host-cell alternative splicing through either intrinsic transcriptional activity or by interacting with components of the host-cell splicing machinery. The majority of EBV-induced alternative splicing events are likely to be a direct consequence of the transcriptional activity of EBV proteins, with interaction with the splicing machinery providing an additional mechanism for regulating host-cell gene expression to drive the activation and proliferation of infected B-cells. EBV EBNA1, EBNA2 and EBNA3 proteins act as transcription factors that modify host-cell transcription [84,85,86,87,88,89,90], and EBNA1, which is critical for EBV genome maintenance, replication and transcription, modulates host-cell hnRNPA1, FOX-2 and SF1 splicing factor expression, resulting in changes in the alternative splicing of 89 cancer-associated genes [90,91].

The EBV alternatively spliced SM protein also drives host-cell gene expression by optimizing RNA nuclear export and stability. EBV SM, which lacks the canonical RS domain, acts in a similar manner to host-cell SR proteins, and alters both EBV and host-cell splicing landscapes. SM achieves this by interacting with the SRp20 host-cell splicing factor to enhance splicing events [92] and accurate RNA-sequence data quantification of individual EBV-induced alternatively spliced isoforms unveil a highly intricate pattern.

#### 3.2.3. EBV Factors and Interaction with Host-Cells

Alternative splicing events induced by EBVs during early infection appear to be both directly and/or indirectly mediated by EBV alternatively spliced EBNA2 and/or EBNA-LP proteins [31]. EBNA-LP interacts with the host-cell pre-mRNA splicing regulator RBM4, which is not only implicated in pre-mRNA splicing but also in translation and RNA silencing, contributing to host-cell activation and proliferation. EBNA-LP acts as an EBNA2 transcriptional coactivator and cooperates with the large multi-protein RBP-Jk complex that mediates transcriptional activation of both cellular and viral promoters [83]. EBNA-LP may also enhance immune evasion by suppressing the innate cell response to viral DNA [93]. EBNA2 trans-activates many cellular and viral genes without directly binding to DNA but by interacting with cellular CBF1/RBP-Jk, PU1, and other transcription factors [94], which, through aberrant alternative splicing, expand the proteomic diversity required for tumor initiation, clonal expansion and progression.

### 3.3. Human Gammaherpesvirus Kaposi’s Sarcoma-Associated Herpesvirus (KSHV, HHV-8)

Kaposi’s sarcoma-associated herpesvirus (KSHV), also known as HHV-8, is a carcinogenic virus identified as the etiological agent of Kaposi’s sarcoma and correlated with other malignancies, including primary effusion lymphoma and multicentric Castleman disease [95,96]. KSHV is largely transmitted sexually and through saliva [97] and exhibits a broad cellular tropism infecting different cell types, including endothelial cells, B cells, epithelial cells, dendritic cells, monocytes and fibroblasts [98].

KSHV belongs to the Herpesviridae family and, like other herpesviruses, has a large, linear double-stranded DNA of approximately 165kb [99], which encodes several proteins expressed in latency and lytic replication phases of the virus life cycle [100]. Control of gene expression is fundamental for KSHV infectivity and oncogenic activity. The latency-associated nuclear antigen (LANA) is a master regulator in establishing and maintaining the KHSV latency phase. This multifunctional 1162 amino acid nuclear protein exhibits potent oncogenic activity. The KSHV latent locus not only encodes LANA but also the fundamental latent phase regulators vFLIP, vCyclin ORF72 and vFLIP (ORF71 or K13), viral miRNAs and Kaposin (K12) [101]. LANA interacts with many host proteins to regulate apoptosis and cell proliferation [102], and targets both p53 and pRB tumor suppressors, contributing to oncogenesis and tumor progression [103]. LANA also stabilizes and promotes the nuclear accumulation of β-catenin, increasing host-cell proliferation and survival [104].

KSHV also alters host-cell RNA splicing through latent genes involved in the regulation of alternative splicing [105]. Specifically, the spliceosome complex C component FAM50A (Family With Sequence Similarity 50 Member A) has been implicated in KSHV-induced cellular transformation through SHP2 alternative splicing, which is a strong mediator and activator of STAT3 in KSHV-transformed cells. STAT3 is involved in many tumor-promoting pathways [105,106].

Extensive analysis of KHSV-infected cells during early infection has also highlighted alternative splicing events, including alternative splicing of IL1β, INSR, IRS2, FOXC2, PRKCA, and SOCS7, although the underlying molecular mechanisms involved remain to be elucidated [107].

Similarities and differences in the oncogenic gammaherpesviruses EBV (HHV-4) and KSHV (HHV-8) are outlined in Table 1.

### 3.4. Hepatitis B, C and D Viruses (HBV, HCV, HDV)

Hepatitis B (HBV), C (HCV) and D viruses are hepatotropic viruses and represent major risk factors in hepatocellular carcinoma (HCC) pathogenesis and progression. These viruses drive hepatocyte transformation via a complex and multifaceted process that involves chronic hepatic inflammation and epigenetic modifications, which subvert cell signalling pathways and promote immune dysfunction [108,109,110,111].

Random integration of the HBV genome into the host-cell genome characterizes HBV as a direct oncogenic agent, leading to mutagenic DNA damage, including deletions, translocations, large inverted duplications, and gene amplifications [112]. Furthermore, site-specific HBV integration can directly activate host-cell oncogenes or eliminate host-cell tumor suppressor genes. HBV proteins also influence host-cell gene expression via epigenetic mechanisms, dysregulating a variety of different oncogenic signalling pathways [108]. HBV-positive HCCs also exhibit extensive splicing alterations, implicating the dysregulation of splicing dysregulation in HCC pathogenesis and progression [113].

#### 3.4.1. HBV and RNA Processing

HBV is a DNA virus of the Hepadnavirus family, with a partially double-stranded circular genome of approximately 3.2 kb. The HBV RNA undergoes extensive alternative splicing, generating at least 20 different alternatively spliced transcripts, the majority of which associate with defective viral reproduction. This indicates that changes in the control of HBV RNA splicing are likely to influence a wide range of viral and host-cell processes in HBV-positive liver disease [114].

The hepatotropic and persistent infectious nature of HBV underpins its involvement in altering hepatocyte gene expression, exemplified by the interaction of HBV proteins with cellular RNA-binding proteins (RBPs) that alter host-cell RNA processing and stability and promote alternative splicing [115]. Indeed, HBV regulation of splicing factor SRSF10 phosphorylation influences HBV RNA levels without influencing splicing [116].

HBVs also reshape the host-cellular microenvironment via ABCF1, HMGA1, RPL28, RBM10, RBM14, and PABPC4, essential for HBV RNA processing during viral replication, triggering metabolic shifts that subsequently modulate gene expression [117].

The HBV alternatively spliced RBM14 and RPL28 proteins act as biomarkers for HBV-positive HCC. RBM14 induces N-methyl adenosine (m6A) modifications that enhance HCC aggressiveness and tumor-promoting Kupffer cells M2-type polarization [118], whereas RPL28 sustains HCC cell proliferation by increasing MDM2 expression, reducing p53 tumor-suppressive function [117,119,120].

#### 3.4.2. HCV Interactions with Infected Cells

In contrast to HBV, the HCV RNA genome encodes a single open reading frame (ORF) that does not undergo alternative splicing, is expressed as a single polypeptide of approximately 3000 amino acids, proteolytically processed into 10 distinct viral proteins and, therefore, does not rely upon the host-cell splicing machinery [121]. The HCV Core protein targets and modulates DDX3 expression and function in alternative splicing, translation, cell cycle regulation and apoptosis [122,123,124]. This indicates that HCV dysregulation of host-cell alternative splicing in HCC is not only a by-product of malignancy but by increasing proteomic diversity also acts as a primary oncogenic driver of tumor initiation, survival and adaptation [18,125].

#### 3.4.3. HBV and HCV-Induced Aberrant Alternative Splicing in HCCs

HBV and HCV-positive HCCs exhibit extensive changes in the alternative splicing patterns compared to normal healthy tissues [126], including alternative splicing of transcription factors, tumor suppressors, and metabolic enzymes, including alternatively spliced isoforms of BCAT2 and ECHDC2 metabolic enzymes, the oncogenic G-protein KRas and the tumor suppressor BRCA1. These isoforms associate with dysregulated splicing machinery and are underscored by high-level expression of hnRNPC splicing factor, which carries a poor prognosis in HCC [127].

The HBV HBx oncoprotein actively modulates cellular alternative splicing in HCCs by interacting with the splicing factor SF1, which is essential for spliceosome assembly. HBx also interferes with cellular signal transduction, transcription, proliferation, and apoptotic pathways, all of which influence HCC progression [128,129]. HBV-positive HCCs also exhibit aberrant HLA-A, HLA-C and IP6K2 alternatively spliced isoforms, involved in immune evasion and tumor cell migration, invasion and metastasis [127].

### 3.5. HDV

HDV, like HCV, is also a single-stranded RNA virus that specifically infects hepatocytes and is now classified as a group 1 carcinogenic virus, capable of directly causing hepatocellular carcinoma [130]. HDV, however, is a defective satellite virus that cannot replicate or cause cancer alone and requires the presence of active HBV infection to survive and propagate [131]. HDV does not undergo alternative splicing of its own genome, which encodes a single open reading frame for the delta antigen (HDAg) [132]. However, alternative splicing is amongst potential HDV oncogenic mechanisms that involve modifications in key signaling pathways involved in fibrosis, epigenetic changes, immune responses and changes in the host-cell proteome. With respect to hijacking and subverting host-cell splicing machinery, HDV genomic RNA interacts with and sequesters the splicing factor SF3b155, a component of the U2snRNP complex essential for early recognition of 3′ splice sites in human gene pre-mRNAs. This interaction alters host-cell alternative splicing, and has been shown to cause abnormal splicing and reduced expression of the tumor suppressor nuclear RNA-binding protein RBM5, which acts within the spliceosome to promote pro-apoptotic alternative splicing of apoptosis-related genes and enhances p53 activity to inhibit cell growth [133,134]. By reducing RBM5 protein levels, HDV-induced alternative RMB5 splicing contributes to early progression in hepatocellular carcinoma [135].

### 3.6. Human T-Cell Leukemia Virus Type 1 (HTLV-1)

HTLV-1 is a member of the Retroviridae family, and causes adult T cell leukemia (ATL), HTLV-1–associated myelopathy or tropical spastic paraparesis (HAM/TSP) [136]. Due to low infectivity as a free particle, this virus requires cell-to-cell contact between T-cells in order to spread, corroborated by significant mother-to-child transmission via breastfeeding, childbirth and in utero, and by sexual transmission [137]. HTLV-1 exhibits specific tropism for CD4+ effector/memory T lymphocytes but can also infect other lymphocytes, dendritic cells and macrophages [138,139]. A defining characteristic is that pro-viral infections that remain latent for decades, and carry an ≈5% risk of eventually developing ATL [140].

The relatively small 9Kb HTLV-1 single-stranded RNA genome encodes structural proteins, functional enzymes and regulatory p8, p12, p13, p30, Tax and bZIP factor (HBZ) proteins [141]. Tax and HBZ are both oncogenic proteins, the effects of which are primarily mediated via protein-protein interactions that alter host gene expression [142,143]. Tax and HBZ share target exons but exert opposite effects on host-cell alternative splicing [144].

#### HTLV-1 Proteins as Modulators of Alternative Splicing

HTLV-1 Tax induces aberrant splicing of genes independent of its effects upon transcription [145]. This largely depends upon activation of the NF-kB transcription factor, through RELA recruitment to intragenic regions, exacerbating physiological processing during gene expression. RELA interaction with DNA results in the recruitment of the DEAD-box RNA helicase DDX17, involved in RNA processing and splicing, increasing alternative splicing events. In addition, Tax binds to the RNA-binding protein U2 auxiliary factor large subunit (U2AF2), consistent with a role in perturbing the spliceosome. In contrast, HBZ, in addition to promoting cell proliferation and transformation, promotes alternative splicing of cancer-associated genes [144,146,147]. The HTLV-1 RNA-binding protein Rex also regulates viral mRNA alternative splicing, transport, viral persistence and dissemination, and these activities are regulated by host-cell hnRNP A1, SRSF1 SF2, nucleolar B-23 and CRM exportin proteins [148,149].

### 3.7. MCPyV Merkel Cell Polyomavirus

Merkel cell polyomavirus (MCPyV), which has a small, nonenveloped, double-stranded circular DNA genome of approximately 5 kb, belongs to the Polyomaviridae family [150]. MCPyV is very abundant in the healthy population, where it persists in latent form as a commensal in the human skin virome. Rarely, in immunosuppressed individuals, the MCPyV genome becomes inserted into the host Merkel cell genome, causing the rare, highly aggressive, neuroendocrine tumor, MCPyV-positive Merkel cell carcinoma (MCC), which represent ≈80% of all MCCs [151].

The MCPyV genome early region encodes a single primary mRNA that undergoes alternative splicing to generate large tumor (LT) and small T (sT) antigens, which target and inactivate pRb and p53 tumor suppressor genes, and both are required for MCPyV-positive MCC pathogenesis and progression [152,153]. This region also encodes alternatively spliced 57 k T antigens and ALTO, providing alternate LT ORFs [150,151]. T-antigen expression in MCPyV-positive MCCs is required for MCPyV-infected Merkel cell proliferation and invasion [151,154,155,156,157,158].

#### Emerging Molecular Mechanisms: Splicing and Oncogenesis

The rising incidence of MCPyV-positive MCC, coupled with poor survival rates, highlights the urgent need for a deeper understanding of the molecular mechanisms underlying this particular tumor pathology. A recent report has detected a novel potentially actionable alternative splice-mediated oncogenic mechanism in MCPyV-positive MCC, characterized by expression of the oncogenic alternative TrkAIII splice variant of the neurotrophin receptor TrkA. This splice variant, originally detected in high-risk advanced stage and metastatic neuroblastomas, exhibits exons 6, 7 and 9 skipping and oncogenic activity in various experimental systems [33], and is induced by polyomavirus SV40 large T-antigen in neuroblastoma cells, further implicating oncogenic polyomaviruses in promoting oncogenic activation of the TrkA receptor tyrosine kinase via alternative splicing. Although the influence of TrkAIII in MCPyV-positive MCC remains to be determined, its expression in advanced-stage and metastatic MCPyV-positive MCCs supports a potentially actionable oncogenic function [32,33,159]. The alternatively spliced MCPyV small t antigen has also been implicated in angiogenesis, invasion and metastasis by promoting expression of the matrix metalloproteinase MMP-9 [160,161].

### 3.8. Human Immunodeficiency Virus 1 (HIV-1)

Human immunodeficiency virus 1 (HIV-1) is a positive-strand RNA virus cause of the immunodeficiency syndrome, also known as AIDS and belongs to the lentivirus family [162]. The primary routes of transmission include mucosal exposure during sexual contact, percutaneous inoculation, and vertical transmission from mother to child [163]. HIV exhibits a specific tropism for T-cells, macrophages, and eventually dendritic cells [164], and utilizes CD4 as its primary receptor, with infections supported by CCR5 or CXCR4 co-receptors [165].

In contrast to other oncogenic viruses, HIV-1 causes malignancy indirectly by inducing immunosuppression, resulting in a permissive environment for opportunistic viral co-infections, and as a consequence is considered to be a group 1 carcinogenic virus rather than an oncovirus. In this regard, HIV-1 infection is not a direct oncogenic driver but significantly elevates the risk of Kaposi’s Sarcoma by facilitating KSHV infection, and is, therefore, a risk enhancer in a multi-step process that predisposes to KSHV infection, rather than a direct oncovirus [166,167].

HIV-1 propagation is also contingent upon hijacking host T-cells’ internal machinery, resulting in alterations in host-cell alternative, corroborated by heightened levels of aberrant alternative splicing in individuals living with HIV/AIDS, which are closely associated with AIDS-associated malignancies, including Kaposi’s sarcoma, non-Hodgkin lymphoma, and cervical cancer [168]. Furthermore, the HIV-1 RNA transcript undergoes extensive alternative splicing, enabling the expression of a vast array of proteins necessary for the HIV life cycle, confirming that HIV-1 also hijacks the host-cell splicing machinery [169].

The characteristics of human carcinogenic viruses are summarized in Table 2.

## 4. Functional Consequences of Oncovirus-Induced Alternative Splicing for Tumor Biology

### 4.1. Oncovirus-Induced Alternative Splicing and Sustained Host-Cell Proliferation

Sustained autonomous proliferation is a central hallmark of cancer, which enables tumor initiation and metastatic progression by promoting an aberrant scenario where cells multiply rapidly and uncontrollably, and do not respond to proliferation-inhibiting signals [170].

The cell cycle is controlled by a finely tuned system of regulatory proteins whose activity is often modulated at the alternative splicing isoform level [171]. In this regard, alternative splicing of oncoviral transcripts can produce viral protein isoforms that drive sustained proliferation by overriding cell cycle checkpoints, exemplified by HR-HPV expression of E6 and E7, and MCPyV polyomavirus expression of large T oncoprotein isoforms that inactivate the tumor suppressors p53 and pRb, removing their capacity to block the cell cycle in cells exhibiting DNA damage [172]. EBV oncovirus uses alternative promoters and alternative splicing to generate different isoforms of the viral oncoprotein LMP1, which include full-length LMP1, produced by double splicing of *ED-L1* pre-mRNA that promotes sustained proliferation by activating MAPK, PI3K/Akt, and STAT signalling pathways, with Akt activation also implicated in aberrant splicing of host-cell transcripts [173]. In contrast, the truncated lytic lyLMP1, generated by single splicing of the *ED-L1A* transcript, negatively regulates LMP1 activity [18,121].

With respect to aberrant alternative splicing of host-cell transcripts by oncoviruses, 14.3.3 proteins are highly conserved molecular scaffolds that bind hundreds of signalling proteins involved in cell cycle control and are expressed as seven alternatively spliced isoforms (α/β, ε,γ,η,σ,θ/T, δ/ζ) that modulate cell cycle progression, often with opposing oncogenic or tumor-suppressive effects. The 14-3-3ζ isoform is oncogenic, whereas 14-3-3σ acts as a tumor suppressor and 14-3-3T promotes degradation of the cell cycle inhibitor p21/Waf1/Cip1. HPV, EBV, HBV, HCV and HTLV-1 oncoviruses promote 14-3-3 alternative splicing, resulting in 14-3-3ζ, 14-3-3ε and 14-3-3 θ/T expression by hijacking RNA-binding proteins and modifying host splicing factors, creating specific intracellular environments that promote host-cell proliferation with transforming potential [18,174,175]. HPV-infected cells also exhibit up-regulated SRSF10 splicing factor expression, leading to alternative BCLAF1-L splicing of the multifunctional nuclear protein BCLAF1 that regulates RNA processing and DNA damage repair. BCLAF1-L acts as a strong oncogenic driver that promotes cancer cell proliferation [47,176]. HBV-infected cells exhibit alternative SREK1L splicing of the splicing regulatory glutamic acid and lysine-rich protein SREK1, which regulates RNA processing and editing, to promote tumor cell proliferation in hepatocellular carcinomas [48], and also exhibit increased expression and alternative splicing of the proliferation-promoting ribosomal protein RPL28, a potential biomarker of HBV-associated disease, inhibition of which inhibits hepatocellular carcinoma cell proliferation [117,177].

The serine threonine kinase Akt also plays a critical role in sustaining cell proliferation [178] and is expressed as Akt1, Akt2 and Akt3 alternative splicing isoforms [179]. Furthermore, Akt activation regulates RNA splicing by phosphorylating components in the histone modification machinery [173]. The high-risk (HR) HPVs 16 and 18 oncoviruses induce aberrant Akt splicing and HR-HPV E6/E7 oncoproteins activate PI3K/Akt/mTOR signalling, resulting in expression of the tumor-promoting Akt3 isoform that enhances host-cell proliferation and survival, which is detected in HPV-positive oropharyngeal squamous cell carcinoma [180]. It is plausible but still hypothetical that oncoviruses, such as HBV, EBV and HT-LV1, known to alter host-cell splicing factors and activate Akt, may do the same, supported by the activation of Akt by the HBV oncoprotein HBx [114]. In contrast, KSHV viral proteins increase the expression of the spliceosome component FAM50A, which induces SHP2 splicing to sustain STAT3 activity, which promotes proliferation and appears to be critical for KSHV-induced transformation [105,106].

EBV oncoviruses also regulate alternative splicing of the NF-YA component of the ubiquitous pioneer nuclear transcription factor NF-Y, altering the ratio of long NF-YAl and short exon 3 skipped NF-YAs isoforms in favour of the NF-YAs isoform that promotes proliferation and rapid cell cycle progression, and is the predominant isoform expressed in EBV-positive stomach carcinomas [181,182]. Clinical data from Cluster 1 hepatocellular carcinomas, frequently associated with HBV or HCV infection and characterised by alterations in hTERT and/or p53, indicate that only the NF-YAs isoform correlates with worse prognosis, indicating that EBV, HBV and HCV facilitate NF-YAs alternative splicing in order to promote rapid proliferation [183]. Furthermore, HDV ssRNA binds and sequesters the spliceosome component SF3b155, resulting in the suppression of RMB5 protein expression via alternative splicing. This promotes sustained growth by reducing the enhancing effect of RMB5 on p53-induced growth suppression [133,134].

With respect to oncogenic polyomaviruses, MCPyV-positive Merkel cell carcinomas express the oncogenic TrkA neurotrophin receptor tyrosine kinase alternative splice variant TrkAIII, which is also induced by SV40 polyomavirus large T antigen in human neuroblastoma cells. TrkAIII in is characterized by exons 6, 7 and 9 skipping, and in contrast to the cell surface full length TrkA receptor, exhibits intracellular retention and accumulation, and promotes primary and metastatic tumorgenicity by exhibiting intracellular ligand-independent cell cycle and stress-regulated activation that activates PI3K/Akt signalling to sustains proliferation and enhance survival [32,33,34], with plausible but still hypothetical potential to further alter host-cell splicing through Akt activation [173].

Oncoviruses also alter SR and hnRNP splicing factor expression and activity to promote sustained proliferation through aberrant alternative splicing of cell cycle cyclins. In this regard, the oncogenic alternatively spliced cyclin D1 isoform, cyclin D1b, characterized by intron 4 retention, increases transforming potential and is often expressed in viral cancers [184], exemplified by HPV regulation of ASF/SF2 splicing factor function [185], which regulates oncogenic cyclin D1b alternative splicing [186].

Group 1 carcinogenic viruses HPV, HBV, EBV, KSHV, HTVL-1 and HIV also transactivate human endogenous retroviral (HERV) sequences within host-cell genomes [187,188,189], and by hijacking the host-cell splicing machinery [23] can promote the expression of host-cell proliferation-promoting HERV proteins, such as alternatively spliced Np9 and Rec proteins from HERV-K pre-mRNAs, and alternatively spliced syncytins from HERV-W [188,190,191,192].

**Figure 3:**
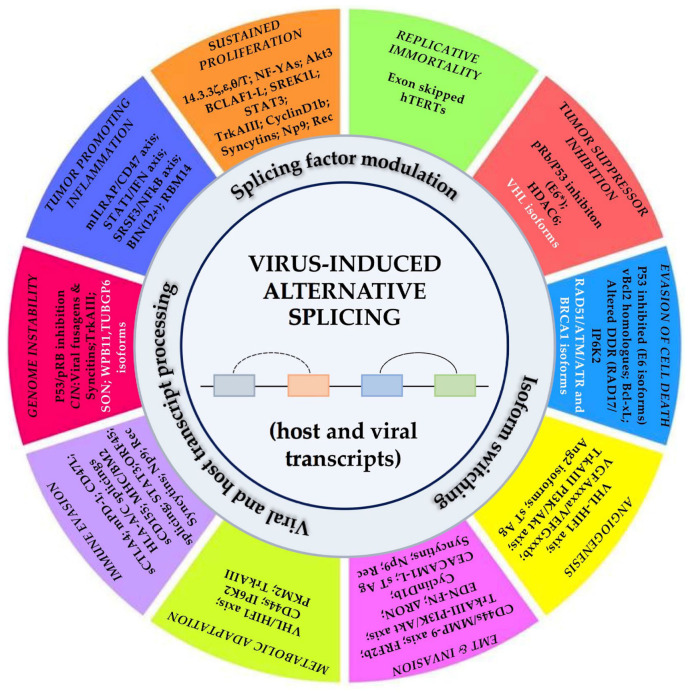
Virus-induced alternative splicing events and their downstream effectors in the 10 hallmarks of cancer, with reported alternative splicing events and downstream effects in black and potential alternative splicing events in white.

It is also apparent that conditions within the normal cellular context may also influence oncovirus-induced transformation, exemplified by epithelial cell EGF production, that influences HR-HPV 16-induced host-cell transformation by promoting alternative splicing of viral transcripts, resulting in E6 expression and alternative E6* splicing and E7 expression, that dysregulate epithelial cell proliferation and promote survival (Figure 3) [193].

### 4.2. Oncovirus-Induced Alternative Splicing and Tumor Suppressor Inactivation

The inactivation of tumor suppressors is a fundamental and defining hallmark of cancer, under the broader concept of evading growth suppression, as tumor suppressors act as brakes to prevent excessive proliferation and ensure genomic integrity [194].

All oncoviral genomes hijack the host-cell splicing machinery to generate multiple viral proteins from a single viral pre-mRNA, expanding their coding capacity and promoting oncogenesis [23]. In doing so, oncoviruses generate alternative splicing viral oncoproteins capable of degrading or inhibiting tumor suppressors. The p53 and pRb proteins are considered to be major tumor suppressors [195] and are major targets of alternatively spliced viral oncoproteins.

HR-HPV E6 oncoprotein targets and inhibits the p53, whereas the E7 oncoprotein targets and inhibits pRb [196]. SV40 poliomavirus large T antigen alternative splicing oncoprotein binds and inhibits both p53 and pRB [197], whereas MCPyV polyomavirus large T antigen binds and inactivates pRB and indirectly inactivates p53-mediated tumor-suppressing pathways [198]. The EBV BZLF1 alternative splicing oncoprotein interacts with and inhibits p53 [199], whereas the EBNA-3C alternative splicing oncoproteins interact with and inhibit the function of both p53 and pRB [200]. The KSHV alternative splicing oncoprotein LANA destabilizes p53 and binds to pRB, inhibiting tumor suppressor functions, whereas KSHV v-IRF-1 and vIRF-3 oncoproteins inhibit p53 function [2,201]. KSHV viral proteins also increase the expression of the spliceosome component FAM50A, which induces SHP2 splicing to sustain STAT3 activity, which promotes survival by negatively regulating p53 expression, and appears to be critical for KSHV-induced transformation [105,106]. The HTLV-1 Tax alternative splicing oncoprotein inhibits p53 by modulating the p300/CBP transcription factor and also represses FBXW7 tumor suppressor, stabilizing the oncogenic transcription factor c-Myc [202,203]. The HBV alternative splicing oncoprotein HBx interferes with p53 function by repressing p53 transcription [204] and the HCV alternative splicing oncoprotein NS5A binds to and degrades p53 [205]. These inhibitory mechanisms effectively allow infected cells to override cell cycle G1/s and G2/M cell cycle checkpoints in DNA-damaged states, promoting proliferation and the subsequent accumulation of genetic mutations.

Almost all oncoviruses also activate the hypoxia response by increasing levels of HIF transcription factors, by stabilizing HIF-1α and HIF-2α, increasing HIF subunit synthesis and/or preventing HIF-1α degradation, even in the presence of oxygen [206,207]. It is highly plausible, therefore, that this may involve oncovirus-induced aberrant splicing of the VHL tumor suppressor, a master regulator of HIF [208], resulting in exon skipping and loss of function VHL isoforms, similar to those described in von Hippel–Lindau disease [209], in addition to targeting the VHL-mediated HIF-1α degradation pathway [210].

HIF transcription factors are also involved in inhibitory alternative splicing of the *HDAC6* gene, resulting in intron-retention, and reduced p53 function by repressing the expression of HDAC6-dependent p53 binding protein 1 and the p53 target gene cell cycle inhibitor P21/Waf1, and by impairing recognition of H4K20me2 and H2AK15ub histone marks induced by DNA double strand breaks and DNA repair [211], unveiling a plausible indirect mechanism for tumor suppressor inactivation through oncovirus-induced, HIF-mediated alternative splicing (Figure 3).

### 4.3. Oncovirus-Induced Alternative Splicing and Replicative Immortality

Replicative immortality is a key cancer hallmark that allows malignant cells to divide indefinitely and bypass the normal “Hayflick” limit of 40 to 60 divisions, which triggers cellular senescence or apoptosis [212]. Cancer cells achieve this primarily by maintaining telomer length via telomerase expression or by the ALT pathway, which prevent chromosome erosion and senescence [213].

Telomer length depends upon de novo telomer synthesis, which in turn depends upon TERT telomerase reverse transcriptase activity. This is also regulated by alternative splicing, which generates inactive or dominant-negative hTERT alternative splicing isoforms, such as the exon 2-skipped alternatively spliced hTERT isoform that undergoes nonsense-mediated decay, which dictates hTERT expression levels and activity [214]. HR-HPV, EBV, KSHV and HTLV-1 oncoviruses all transcriptionally activate the *hTERT* gene with important effects on telomeric regulation and function to immortalize and increase the proliferative capacity of infected cells [215]. This indicates that some oncoviruses, by hijacking host-cell splicing, promote and maintain hTERT function by overriding host-cell expression of non-functional hTERT alternative splicing isoforms, to achieve replicative immortality (Figure 3).

### 4.4. Oncovirus-Induced Alternative Splicing and Evasion of Cell Death Mechanisms

Evasion of cell death mechanisms is crucial in oncogenesis as it allows genetically damaged, abnormal cells to survive, proliferate, and accumulate further mutations rather than being eliminated. This process, primarily involving evading apoptosis, and is a fundamental hallmark of cancer, enabling tumor initiation, progression, metastasis, and resistance to therapy [216].

Unlike lytic viruses, such as influenza, oncogenic viruses replicate using a strategy of persistence without immediate host-cell killing, and either integrate their genetic material into the host genome or exist as stable circular episomes that co-opt cellular division machinery [217]. Some (KSHV and EBV) oncoviruses enter latent states during which only a small set of genes is expressed to maintain the viral genome and avoid apoptosis by evading immune detection. Viral episomes within the nucleus replicate once per cell cycle, timed perfectly with cellular DNA replication. Oncogenic viruses are also equipped with specialized oncoproteins that actively protect against cellular programmed death mechanisms. This is exemplified by the HPV genome alternatively spliced E6 oncoprotein, which inhibits and degrades the pro-apoptotic host-cell tumor suppressor p53 [218]. Furthermore, HPV E6, E6*I and E6*II spliced isoforms not only decrease p53 activity but also differentially interact with p53 to regulate its function [219]. Another example of apoptosis evasion through viral genome alternative splicing is the expression of EBV vBcl2 homolog alternative splice variants BHRF1 and BALF1, and KSHV encoded vBcl-2, that prevent apoptosis by inhibiting pro-apoptotic BIM, BID, PUMA and BAK proteins and/or by boosting the expression of IAPs that inhibit pro-apoptotic caspase activation or by activating pro-survival MAPK/PI3K/Akt signalling [220,221]. KSHV viral proteins increase the expression of the spliceosome component FAM50A, which induces SHP2 splicing to sustain STAT3 activity, resulting in cell death evasion by down-regulating p53 expression and increasing the expression of Bcl-2, Bcl-xL, Mcl-1 and IAP-2 [105,106,222]. HBV-positive hepatocellular carcinomas also exhibit increased expression and alternative splicing of the ribosomal protein RPL28, which inhibits apoptosis by regulating the p53 pathway [117,177]. Furthermore, ssRNA of the subviral satellite carcinogenic virus HDV binds and sequesters the spliceosome component SF3b155, which suppresses RMB5 protein expression via alternative splicing. This reduces the influence of RMB5 on pro-apoptotic alternative splicing of apoptosis-related genes [133,134].

EBV alternative splicing of LMP isoforms also results in activation of the PERK/ATF4 arm of the host-cell ER stress survival response, promoting proliferation and reducing stress-induced cell death [223].

With respect to the involvement of oncovirus-induced host-cell alternative splicing in apoptosis evasion. Oncoviruses that activate PI3K/Akt signalling either directly or indirectly and induce the alternatively spliced tumor-promoting wild type Akt3 isoform would also be expected to protect cells against apoptosis [224], consistent with the pro-survival role of PI3K/Akt pathway activation [225].

The tumor suppressor RBM5 which functions primarily as a splicing regulator and tumor suppressor, and controls proliferation and apoptosis by regulating the alternative splicing of the Fas receptor, cFLIP and Caspase-2, favouring pro-apoptotic isoforms, is subject to HDV oncovirus-induced alternative splicing, resulting in the expression of isoforms with reduced function that are involved in liver disease progression and transition into hepatocellular carcinoma. HDV RNA regulates this process by interacting with the splicing factor SF3B155, disrupting normal RBM5 splicing, resulting in inclusion or exclusion of specific exon cassettes, to generate unproductive mRNA variants that are either degraded or produce non-functional isoforms that promote proliferation and survival [135].

Oncoviruses also manipulate the host-cell splicing machinery to favour expression of anti-apoptotic long Bcl-xL over the pro-apoptotic short Bcl-xS isoform of the *Bcl-x* gene [226], and oncovirus-positive tumors frequently display a shift in the Bcl-xL/Bcl-xS equilibrium in favour of the anti-apoptotic Bcl-xL isoform [23,227,228]. In this regard, a major KSHV/HHV-8 locus is involved in BcL-xL expression and prevention of endothelial cell apoptosis [229]. EBV oncoviruses up-regulate Bcl-xL expression through LMP-2A and BARF1 proteins [230], HR-HPV-16 promotes SRSF10 splicing factor expression resulting in enhanced Bcl-xL expression in cervical cancer [15,231], and HTLV-1 oncovirus Tax protein is responsible for Bcl-xL expression in transformed T-cells [232].

As stated in the previous section, the oncogenic TrkAIII splice variant expressed in MCPyV-positive Merkel cell carcinomas, and induced by SV40 polyomavirus large T antigen in neuroblastoma cells, exhibits intracellular activation and activates pro-survival PI3K/Akt signalling, increasing the expression of anti-apoptotic long-form Bcl-xL and Mcl-1L alternative splicing isoforms [32,33,34,233], which, combined with sustained proliferation, increase both tumorigenic and metastatic capacity in neuroblastoma models. TrkAIII also promotes a more stem cell-like tumor cell phenotype with enhanced resistance to the genotoxic chemotherapeutic agents cisplatin and doxorubicin, and the highly cytotoxic, development and doxorubicin-regulated, extra-short NF-YAx alternatively spliced isoform of the ubiquitous pioneer transcription factor NF-Y component NF-YA, confirming that the polyomavirus-associated TrkAIII oncoprotein facilitates host-cell evasion of cell death mechanisms [32,33,234,235].

With respect to evading cell death induced by DNA damage, which is invariably induced by oncoviruses, this is also achieved by modifying specific splicing changes involved in DDR-regulated DNA repair, cell cycle checkpoints and apoptosis responses (DDR) [23,236,237]. HTLV-1 and HR-HPVs alter alternative splicing to disrupt signalling from the serine/threonine kinase ataxia telangiectasia-mutated (ATM), which is activated by double-strand DNA breaks, causing DDR defects. HTLV-1 Tax and HBZ proteins interact with U2AF2 splicing factor to alter alternative splicing of the ATM gene, causing exon inclusions and exclusions that produce non-functional ATM isoforms that often lack the C-terminal kinase [144,238]. It is plausible that HR-HPV E6 and E7 proteins may also do the same [43].

It is also plausible that oncoviruses may also promote alternative splicing of the DDR serine/threonine kinase ataxia-talengiectasia-Rad3-related (ATR), which is activated by single-stranded DNA [237,239], by modifying the activity and expression of SRSF1 and hnRNP1 splicing factors, promoting aberrant ATR isoforms, such as cancer associated ATR isoform 2, characterized by exon 6 skipping, which fail to activate checkpoint kinase CHK1 [240,241]. HR-HPVs also promote changes in the capacity of ATR to activate CHK1 in HPV positive cervical cancer, by promoting a splice variant switch in claspin isoforms, allowing DNA-damaged infected cells to circumnavigate the G2/M checkpoint [242,243].

In addition to coordinating DNA repair, cell cycle arrest and apoptosis, ATM and ATR also influence alternative splicing by directly phosphorylating SRSF, such as SRSF1 and related RNA-binding proteins involved in RNA processing, particularly under stress conditions [244]. This highlights an additional potential indirect mechanism through which oncoviruses may modify alternative splicing by promoting the expression of dysfunctional ATM and ATRs, in addition to apoptosis inhibitors, to sustain viral replication, which depends upon the proliferation and survival of DNA-damaged infected host cells.

It is also plausible that oncoviruses, by altering the SRSF/hnRNP splicing factor equilibrium to corrupt the host-cell splicing machinery and by disrupting the spliceosome to promote aberrant splicing that favours survival over DNA repair, may modify the splicing of DNA repair genes such as RAD17, expressed as 4 alternative splice variants with different potential functions [245], RAD51 isoforms with altered function [246] and RIF-1, that undergoes DNA damage-induced alternative splicing into RIF1-L and RIF-1 S isoforms with different functions in the DDR [247], but this remains hypothetical. EBV oncoviruses also promote the methylation of CpGs and introduce histone modifications near splice sites, which promote alternative splicing patterns that down-regulate the expression and activity of DNA repair genes [248].

HR-HPVs 16 and 18 promote E2F1-mediated expression of the splicing factor SRSF10 [46], which is involved in the DDR [231]. SRSF10 phosphorylation promotes splicing activation, whereas de-phosphorylation promotes splicing repression [249], and DNA damage induces SRSF10 de-phosphorylation, re-configuring the alternative splicing of gene transcripts involved in DNA repair and apoptosis. Indeed, oxaliplatin-induced SRSF10 de-phosphorylation promotes ∆9-10 BRCA1 and Bcl-xL alternatively spliced isoform expression by interacting with casein kinase 1ε, enhancing host-cell survival within a context of DNA homologous recombination [105,231]. Increased SRSF10 expression, combined with protein phosphatase-1-mediated SRSF10 de-phosphorylation, increases genomic instability by promoting expression of alternatively spliced BRCA-1 and Bcl-xL isoforms, which contribute to viral oncogenesis and metastatic progression by impairing the DDR and enhancing survival, proliferation and the accumulation of mutations in DNA-damaged host cells (Figure 3) [236,250,251].

Alternative splicing of the pro-apoptotic protein inositol hexakisphosphate kinase 2 (IP6K2) also characterizes HBV-positive hepatocellular carcinomas, with potential to disrupt p53-induced apoptosis [127,252].

### 4.5. Oncovirus-Induced Alternative Splicing and Tumor Angiogenesis

Angiogenesis is a physiological process of developing new blood vessels from pre-existing blood vessels, and is a critical hallmark of cancer, which is hijacked by tumors to ensure a supply of oxygen and nutrients required for clonal expansion at both primary and metastatic sites, and facilitates the metastatic process by forming immature, disorganized, easily penetrated tumor vasculatures [253,254,255].

Oncoviruses modulate angiogenesis both directly and indirectly to promote initial tumor growth and metastatic progression by reshaping host-cell alternative splicing of angiogenesis-related genes [18,23,168,256]. Vascular Endothelial Growth Factor A (VEGFA) is a critical angiogenic mitogen that orchestrates angiogenesis by ligating the VEGF receptor VEGFR2, to induce the proliferation of endothelial cells in pre-existing de-stabilized blood vessels, which is required for angiogenesis [257,258,259]. The KSHV oncovirus promotes angiogenesis through the viral G protein-coupled receptor (vGPCR), which induces VEGFA expression and activates pro-angiogenic signalling pathways [168,260,261]. Oncoviruses also promote angiogenesis by corrupting the host-cell splicing machinery, altering alternative splicing in favour of pro-angiogenic VEGFAxxxa over anti-angiogenic VEGFAxxxb isoforms, through differential use of proximal and distal splice sites at the end of exon 8 [256,262]. Oncoviruses that exploit VEGFA and corrupt alternative splicing to ensure a pro-angiogenic VEGFAxxxa/VEGFAxxxb equilibrium include HBV, through the alternatively spliced HBx protein, which stabilizes HIF-1, up-regulating VEGFA expression and angiogenesis during liver carcinogenesis [263,264,265]. HCV core protein also promotes VEGFA expression via the NF-κB/HIF1α axis [266] and HR-HPV alternatively spliced E5, together with E6 and E7 proteins, promote VEGFA expression, and E6 is a specific activator of the VEGF promoter [267]. The EBV late membrane protein-1 (LMP1) also up-regulates VEGFA expression, as do KSHV alternatively spliced viral vFLIP, K1 and vIRF3 proteins. Viral corruption of the host-cell splicing machinery, frequently driven by dysregulated SRSF1 and SRPK1 splicing factors expression and activity, combined with induction of VEGFA expression, ensures a pro-angiogenic VEGFAxxxa/VEGFAxxxb equilibria in viral cancers [268]. KSHV viral proteins also increase the expression of the spliceosome component FAM50A, which induces SHP2 splicing to sustain STAT3 activity, which also promotes angiogenesis by up-regulating VEGFA expression [105,106].

As stated previously, the MCPyV-induced oncogenic alternative TrkAIII splice variant also promotes a pro-angiogenic phenotype by activating the PI3K/Akt signalling, resulting in increased expression of pro-angiogenic MMP-9 and VEGFA and reduced expression of the angiogenesis inhibitor thrombospondin-1, adding angiogenesis to sustained proliferation and enhanced resistance to cell death mechanisms to TrkAIII’s oncogenic repertoire [32,33,34,269]. The alternatively spliced MCPyV small T antigen (sT) also activates MMP-9 expression [160,252].

Oncoviruses also elevate host-cell expression of the angiogenic non-mitogen angiopoietin-2, which is a pre-requisite for angiogenesis, and competes with angiopoetin-1 ligation of vascular cell Tie-2 receptors to destabilize pre-existing blood vessels [270]. Although, there is no evidence linking oncoviruses to the generation of functionally different alternative splicing angiopoetin-2 isoforms [271], the carcinogenic HIV-1 virus promotes alternative splicing of angiogenesis-related genes, including angiopoetin-2 [168] and nearly all oncoviruses activate or increase HIF-1 expression which, within a context of oncovirus-dysregulated host-cell splicing, may not only promote angiopoetin-2 expression but also aberrant splicing (Figure 3) [207,272].

### 4.6. Oncovirus-Induced Alternative Splicing, EMT and Tumor Invasion

During tumor progression, stationary primary tumor cells gradually lose cell-to-cell adhesion and basal-apical polarity and transform into mesenchymal/stem cell-like cells that acquire migratory/invasive behaviour that underpins metastatic progression, referred to as epithelial–mesenchymal transition (EMT) [273].

Amongst the many changes that characterize EMT, increased expression of the hyaluronan oligosaccharide receptor CD44 is accompanied by an alternative splicing switch from CD44v to CD44s isoforms, which promote self-sustaining autocrine motility through CD44s ligation of hyaluronan oligosaccharides produced by the same cell. This interaction induces PI3K/Akt signalling and links CD44s to the cytoskeleton, facilitating tumor cell migration and invasion [274,275]. Oncovirus manipulation of host-cell splicing machinery promotes EMT, invasion and migration, by increasing CD44 expression and alternative splicing. HPV E6 and E7 proteins trigger EMT and promote CD44s isoform expression. HBV and HCV, which promote cancer stem cell phenotypes, also promote abnormal CD44s alternative splicing [276,277,278], EBV LMP1 protein is drives CD44s expression [279], KSHV alters RNA binding protein activity to increase CD44s expression [280], and human onco-modulatory cytomegalovirus infection observed in gliomas associates with high CD44s expression and stemness [281]. CD44s also acts as a docking site for matrix metalloproteinases such as MMP-9, localizing proteolytic enzymes to the cell surface, facilitating ECM degradation and tumor invasion [282]. Therefore, oncoviruses that promote host-cell CD44 expression and CD44s alternative splicing, drive EMT and tumor stemness, increasing migratory and invasive behaviour, which underpin metastatic progression [275,283,284,285].

EMT is also promoted by the HR-HPV 16 E5 protein, which down-regulates the expression of the FGFR2 splice variant FGFR2b, switching expression to the FGFR2c isoform, which inhibits FGFR2 activity and promotes EMT [286,287]. It is plausible that expression of the oncogenic TrkAIII splice variant in MCPyV-positive Merkel cell carcinomas, also induced by SV40 large T antigen in neuroblastoma cells [33,269], may also promote EMT and invasive behaviour, as TrkAIII promotes a more stress-resistant stem cell-like phenotype, and enhances tumor cell matrix metalloproteinase MMP-9 expression [34,160], but this remains to be verified.

Oncoviruses also hack the host-cell splicing machinery to generate the alternatively spliced extra domain A fibronectin (EDN-FN) isoform, which promotes lymphangiogenesis and enhances metastatic potential by remodelling the extracellular matrix, facilitating EMT and invasion, and by reorganizing interactions between extracellular matrix components, and tumor cell integrins and cytoskeleton [288,289,290].

Oncoviruses, such as HPV, that disrupt the splicing factor equilibrium by reducing SRSF2 expression or activity also promote *RON* exon 11 skipping, resulting in expression of the ∆RON isoform of this receptor tyrosine kinase, which exhibits constitutive activity and drives cell motility [291].

Oncoviruses, such as HPV, EBV and KHSV, are also closely associated with p-regulating or alternative splicing of the highly oncogenic cyclin D1b alternative spliced isoform of cyclin D1, by promoting use of an alternative splice site in exon 4, introducing a premature stop codon. This isoform is significantly more effective at driving cellular transformation than canonical cyclin D1a and is also a potent promoter of tumor cell invasion, due to omission of Threonine 286, which regulates degradation and promotes nuclear accumulation. HTLV-1 Tax and HBZ proteins also regulate cyclin D1 transcription and splicing, and increased expression of the cyclin D1b alternative spliced isoform, which characterizes cancers caused by HBV, HPV and EBV oncoviruses [184,185,186].

HPV alternatively spliced E6* splice variant proteins also directly drive the expression and activity of matrix metalloproteinases, including MMP-9, which promotes EMT and tumor invasion [160,292].

Although not specifically reported in the literature, it is plausible but still hypothetical that oncoviral corruption of the host-cell splicing machinery may also promote expression of the MBD1a alternative spliced isoform of the epigenetic regulator methyl-CpG binding domain protein 2 MBD2, by mimicking the hypoxia response [207,293]. MBD1a binds to methylated DNA to regulate gene expression by recruiting the NuRD complex to methylated CpG islands, promoting chromatin compaction, gene silencing and specifically facilitates tumor invasion and metastasis [294].

HPV, EBV, MCPyV, HTLV-1 and KSHV oncoviruses also corrupt the host splicing machinery to promote expression of the exon 7 included long-form CEACAM1-L isoform, potentially by altering the equilibrium between hnRNP L, hnRNP A1 and hnRNP M, which regulate CEACAM 1 splicing. This isoform dysregulates CEACAM1 involvement in the immune checkpoint, and promotes tumor invasion and metastasis [295]. As an alternative invasion and metastasis-promoting mechanism, KSHV viral proteins, by increasing spliceosome component FAM50A expression, induce SHP2 splicing, which sustains the activity of STAT3. Sustained STAT3 activity promotes invasion by enhancing the expression of the matrix-degrading MMPs, MMP1, MMP-2 and MMP-9, and by modulating Rac1 activity to maintain directional persistence during migration [105,106].

MCPyV alternatively spliced small T antigen (sT) activates expression of the invasion-promoting matrix metalloproteinase MMP-9, which is also induced by the oncogenic alternative TrkAIII splice variant, expressed in MCPyV Merkel cell [32,33,34,160,161,269].

As stated in Section 3.1, group 1 carcinogenic viruses also transactivate human endogenous retroviruses (HERVs) within host-cell genomes, and by hijacking the host-cell splicing machinery promote the expression of alternatively splicing HERV-K Np9 and Rec and HERV-W syncytins, which promote EMT, migration and invasion (Figure 3) [190,191,296,297].

### 4.7. Oncovirus-Induced Alternative Splicing and Tumor Metabolic Reprogramming

The “Warburg effect” is a metabolic adaptation through which cancer cells produce energy through high rates of glycolysis, followed by lactic acid fermentation, even in the presence of oxygen, rather than from mitochondrial oxidative phosphorylation. This metabo-type not only ensures sufficient levels of energy but also a source of carbon for biosynthetic pathways required for tumor growth [298].

HPV, HBV, HCV, EBV and KSHV oncoviruses and the onco-modulatory virus HCMV (see Section 3.3) promote CD44 expression and CD44s alternative splicing, which, in addition to promoting EMT and tumor invasion, also strongly influences tumor cell metabolism by promoting a highly glycolytic metabolism, leading to increased glucose consumption, ATP and lactate production important for tumor progression [275,299]. Oncoviruses also frequently drive the alternative splicing of pyruvate kinase pre mRNA to favour expression of the PKM2 over PKM1 isoform through differential exon 9 or exon 10 inclusion, respectively. This switch alters PK enzyme kinetics, promoting the “Warburg effect”, resulting in the accumulation of intermediate glycolytic metabolites, which enter the pentose-phosphate pathway for biosynthesis of ribose 5-phosphate for DNA and RNA synthesis and the aromatic amino acid precursor erythrose 4-phosphate to enable rapid proliferation [300,301]. HR-HPV and EBV do this by upregulating hnRNP A1/A2, PTB and SRSF3 splicing factor expression, which bind to the flanking regions of *PKM* mRNA exon 9, forcing skipping and the inclusion of exon 10 (*PKM2*) [302]. HPV E7 and EBV LMP1 proteins also activate the c-MYC oncogene, which directly promotes PKM2 splicing, and the KSHV protein ORF57 modulates splicing machinery with a similar outcome [301]. In addition, the HPV E7 protein binds to PKM2, driving its dimerization rather than tetramerization, which lowers enzyme activity and increases the accumulation of glycolytic intermediates, such as fructose Bisphosphate, essential for amino acid synthesis and NADPH biosynthesis [301]. HTLV-1 Tax and HBZ proteins also promote splicing events relevant to tumor metabolism, including mutually exclusive PKM exon from resulting in PKM2 expression, and HBZ interacts with the RNA binding protein AICF to regulate metabolic enzymes’ alternative splicing, promoting infected cell maintenance within low glucose microenvironments [144]. Alternative splicing of the high energy signaling, mitochondrial function maintaining protein inositol hexakisphosphate kinase 2 (IP6K2) also characterizes HBV-positive hepatocellular carcinomas influencing tumor metabolic adaptation [127].

With respect to polyoma oncoviruses, the oncogenic alternative TrkAIII splice variant, expressed in MCPyV-positive Merkel cell carcinomas and induced by polyoma oncovirus SV40 large T antigen in neuroblastoma cells, promotes stress-induced glycolytic adaptation in neuroblastoma cells by translocating into mitochondria under conditions of ER stress, where it undergoes cleavage-activation. This results in tyrosine phosphorylation of mitochondrial pyruvate dehydrogenase kinase PDK, inhibition of oxidative phosphorylation, and the promotion of glycolysis and lactate production (Figure 3) [303].

### 4.8. Oncovirus-Induced Alternative Splicing in Tumor Immune Evasion

Malignant cells must actively hide from, suppress, or trick the host immune system to survive, grow and form tumors. Without these traits, immune cells would naturally recognize and destroy abnormal cells on the way to neoplastic transformation. 

Oncoviruses, by hijacking the host-cell splicing machinery, create and maintain immunosuppressive TMEs to promote immune evasion, by enhancing the expression of an alternative spliced soluble decoy receptor that allows infected cells to evade immune destruction. HR-HPVs 16/18 positive oral and head and neck cancers, which exhibit virus-modified alternative splicing, express higher levels of the soluble decoy sCTLA4 isoform, which suppresses T-cell activation by binding CD80/CD86 ligands on antigen-presenting cells, in a manner similar to membrane-bound CTLA4 [304].

In contrast, oncoviruses also alter *PDCD1* expression and alternative splicing in favour of the membrane-associated rather than soluble PD-1 isoform, driven by exon 3 exclusion, favouring inhibition of anticancer immunity by ligating PD-L1 and PD-L2 receptors on T lymphocytes, rather than restoring tumor immunity through the soluble PD1∆3 isoform, which inhibits PD-1/PD-L1 interaction [305,306]. PD-1 expression is also promoted by HPV E6 protein, which, by stabilizing HIF-1α [307,308], enhances PD-1 expression in the HPV positive cervical cancer immune micro-environment [307].

Another critical immune checkpoint protagonist subverted by oncoviruses is expression of the full-length CD47 isoform, a transmembrane cell surface protein that prevents cells from being destroyed by macrophages [309]. Dysregulation of splicing by HR-HPV E6 and E7 proteins, by enhancing splicing factor SRFS10 expression, increase the membrane-associated isoform of interleukin-1 receptor accessary protein (IL-1RAP), by modulating the alternative termination signal in exon 13, resulting in full-length CD47 expression [310], this splicing switch from micro exon-skipped CD47S to the full length CD47L isoform is considered to be more effective at promoting an immunosuppressive “don’t eat me” microenvironment [311].

Alternative splicing of the cell surface immunoglobulin-like adhesion protein Necl-5/CD155 also plays a significant role in tumor immune evasion and is expressed as 2 membrane-bound (α and δ) and 2 soluble (β and γ) splice variant isoforms. Soluble sCD155 binds the co-stimulatory receptor DNAM-1 (CD226) on NK and T cells, blocking interaction with tumor cell membrane-bound CD155, inhibiting NK and T-cell-mediated anti-tumor immunity [312,313]. It is plausible, therefore, that oncoviral promotion of CD155 expression and alternative splicing [23,314] may extend to sCD155 isoform expression, but this remains to be verified.

In HBV-positive hepatocellular carcinomas, specific splicing events have been reported in the primary antigen-presenting proteins HLA-A and HLA-C, promoting immune evasion [127].

It is also plausible that oncovirus-induced alternative splicing may extend to *NUMB* exon 9 inclusion transcripts that promote immune suppression by reducing T cell and macrophage infiltration, and are frequently up-regulated in tumors [315]; membrane-bound immune checkpoint inhibitory glycoprotein CD200L isoforms that suppresses myeloid cell activation by interacting with the inhibitory receptor CD200R1, and BM2 isoforms that prevent T cell activity and associate with viral infections and cancer, which together would promote immunosuppressive immunologically “cold” tumors [191,192,193,194,316,317], but this is still hypothetical.

Oncogenic viruses exploit alternative splicing and other regulatory strategies affecting both viral and host genes to bypass immune surveillance. The EBV EBNA1 protein, essential for viral genome persistence, contains a central glycine–alanine repeat (GAr) domain that suppresses mRNA translation and thereby limits proteasome-dependent processing and MHC class I antigen presentation, allowing infected cells to escape detection by cytotoxic T lymphocytes [18,23,318,319]. Similarly, KSHV targets host-cell splicing machinery to deactivate the innate immune response. The viral protein ORF45 interacts with IRF7 and acts as an alternative substrate for TBK1 and IKKε, blocking IRF7 phosphorylation and activation and suppressing type I interferon production. In parallel, alternative splicing of IRF7 itself has been shown to generate isoforms with distinct abilities to drive IFN-I responses, providing an additional layer of regulation of antiviral signalling [320,321]. KSHV viral proteins also increase the expression of the spliceosome component FAM50A, which induces SHP2 splicing to sustain STAT3 activity, which promotes immune evasion by modulating tumor cell secretion of immune-suppressing cytokines [105,106]. HBV also interferes with key innate signalling components, and its HBx protein inhibits pattern-recognition receptor pathways, including STING-dependent signalling, dampening type I interferon responses and preventing efficient detection of infected or transformed hepatocytes [322,323].

As stated in previous sections, group 1 carcinogenic oncoviruses also transactivate HERVs within host-cell genomes and, by hijacking the host-cell splicing machinery, promote the expression of immunosuppressive alternatively spliced products from the HERV gene env, such as HERV-W syncytin-1 and Env59, and HERV-K Np9 and Rec (Figure 3) [188,190,192].

### 4.9. Oncovirus-Induced Alternative Splicing and Tumor Promoting Inflammation

Tumor-promoting inflammation is another key hallmark of cancer, where chronic, non-resolving immune and inflammatory responses within the TME actively fuel cancer development rather than fighting it. This results in cancer progression by facilitating DNA damage, angiogenesis, cellular proliferation and progression to metastatic disease [324].

All oncoviruses induce chronic tumor-promoting inflammation by establishing persistent infections that cause immune and inflammatory cell activation. With respect to inflammatory responses, these are then modified to promote tumor progression by mechanisms that include changes in the expression and function of splicing factors in favour of pro-inflammatory mediator isoform expression. HPVs promote SRSF10 splicing factor expression, which promotes mIL1RAP alternative splicing by modulating the exon 13 alternative terminator, resulting activation of dual pro- and anti-inflammatory transcription factor NF-kB [325], resulting in increased expression of CD47 “don’t eat me” glycoprotein, preventing macrophage phagocytosis of infected cells [46]. On the other hand, EBV SM oncoprotein acts as a splicing factor to modulate STAT1 splicing, a critical component of anti-viral INF signalling, favouring persistent infection within inflammatory TMEs [326]. It is plausible that increased SRSF10 expression in HPV-infected cells may also induce an alternative Bin1(12+) splice variant of the bridging integrator protein BIN1 that promotes the pro-tumorigenic macrophage M2 phenotype, and by interacting with the annexin A1 (ANXA1) protein, promotes an inflammation-suppressing TME by mediating the effects of glucocorticoids [49,327]. In hepatocellular carcinomas, HBV infection promotes alternative splicing of the regulator RNA binding motif protein 14 (RBM14) to promote Kupfer cell M2 phenotype [117,118].

By manipulating host-cell splicing mechanisms, it is also plausible that oncoviruses that increase the expression and activity of the splicing factor SRSF3, which inhibits NF-kB, may also increase the expression of aberrant alternative spliced isoforms that disable macrophage pro-inflammatory signalling and promote the immunosuppressive pro-tumor M2 phenotype (Figure 3) [328,329,330], but this remains to be verified.

### 4.10. Oncovirus-Induced Alternative Splicing, Genomic and Chromosome Instability

Genetic instability is a fundamental hallmark and enabling characteristic of cancer, allowing cells to acquire the mutations and chromosomal aberrations that fuel tumor progression, heterogeneity, adaptability and therapeutic resistance. By accelerating genetic changes, instability enables tumors to evolve, evade growth suppression and acquire the genetic make-up required to achieve all of the cancer hallmarks that eventually lead to metastatic disease [331].

Within this context, cancers driven by oncoviruses accumulate lower total numbers of somatic mutations compared to non-viral, carcinogen-driven cancers due primarily to the fact that they are driven by viral oncoproteins rather than accumulated genetic errors [332,333]. However, cancers driven by oncoviruses do exhibit considerable chromosomal instability, comprised of polyploid and aneuploid states, caused by virus-induced cell fusion [334] or by aberrant centrosome duplication [335,336,337].

Host-cell fusions that result in chromosomal instability in oncoviral tumors are either driven by viral fusion proteins (fusogens), formed by hijacking the host-cell splicing machinery [23] to produce alternative splicing fusogen proteins from viral pre-mRNAs [338]. In addition, as stated in previous sections, group 1 carcinogenic viruses also transactivate host-cell HERVs, and by hijacking the host-cell splicing machinery promote the expression of host-cell fusogens, such as syncytin-1 and syncytin-2 from HERV-W [188,190,192], driving cancer cell fusions by ligating ASCT2 (syncytin-1) or MFSD2A (syncytin-2) receptors, resulting in chromosomal instability [339,340].

With respect to centrosome involvement in chromosomal instability, accurate centrosome duplication is fundamental for bi-polar mitotic spindle formation and accurate chromosome separation in normal cells, and is dysregulated in oncovirus centrosomes, resulting in aberrant centrosome numbers [337,341,342,343,344,345]. Accurate centrosome duplication involves CEP135 mini and short form and SADB serine/threonine kinase alternative splicing and SON RNA binding factor and SF3B14 splicing factor, potential targets for oncoviral dysregulation [346,347,348,349]. Cells with aberrant numbers of centrosomes form multi-polar or pseudo-polar mitotic spindles during cell division, resulting either in mitotic collapse-induced polyploidy or aneuploidy due to aberrant chromosome separation [350,351]. The pre-mRNA splicing factor WPB11 is crucial for pre-mRNA splicing of tubulin gamma complex-associated protein 6 (TUBGCP6), a γ-tubulin component essential for centrosome maturation [352]. SON splicing factor is required for pericentrin (PCNT) and CEP131 mini splicing, involved in centriolar satellite organisation and procentriole assembly [347]. SF3B14 splicing factor is involved in TUBGCP splicing and is required for centrosome regulation and mitotic chromosome alignment [348]. Since disruption of these splicing events leads to defects in centrosome function and duplication [353], and oncoviruses manipulate the host-cell splicing machinery and oncoviral tumors exhibit centrosome amplification, it is highly likely that oncoviruses promote centrosome duplication by dysregulating these critical splicing events [347,350,351], but this remains to be verified.

With respect to oncogenic polyoma viruses, the oncogenic TrkAIII alternative splice variant is expressed in MCPyV-positive Merkel cell carcinomas and is induced by polyomavirus SV40 large T antigen in neuroblastoma cells. In neuroblastoma cells, TrkAIII exhibits intracellular activation and retrograde microtubule-mediated transport back to centrosomes, resulting in polokinase-4 tyrosine phosphorylation, centrosome amplification and chromosomal instability [34,354]. This unveils a plausible hypothetical mechanism through which MCPyV may enhance chromosome instability in MCPyV-positive Merkel cell carcinoma.

## 5. Therapeutic Perspectives

### 5.1. Vaccines

Given the complex nature of oncoviral cancers, in terms of rewiring host-cell signaling when a particular therapeutic agent or the immune system blocks a particular pathway [355] unveils a “Mafia style” playbook by which oncoviruses takeover control of the cell by activating redundant survival mechanism, evade pathophysiological “law” by protecting the “territory” the TME against immune attack and bypass “roadblocks” by triggering alternative growth and survival pathways when a specific pathway is inhibited. It is obvious, therefore, that vaccines against that oncovirus have the greatest potential to lower the global viral-cancer burden, already demonstrated by the major impact of vaccines against HBV and HR-HPVs in preventing liver and cervical cancers [356].

### 5.2. Targeting Oncoviral Proteins

With respect to strategies to treat established oncoviral tumors, an obvious approach would be to directly target the viral oncoproteins involved in inactivating tumor suppressors and tumor-suppressing pathways and hijacking the host-cell splicing machinery to activate host-cell oncogenes and oncogenic signaling pathways through aberrant alternative splicing. In this regard, strategies that directly target HR-HPV E6 and E7 oncoproteins in cervical cancers, HBV HBx proteins in liver cancers, EBV latent membrane protein-1 in B-cell lymphomas, HTLV-1 Tax and HBZ proteins in HTLV-1 induced cancers, and T antigens in MCPyV-positive Merkel cell cancers, through RNA interference and CRISPR technologies, are under intensive investigation and development [357,358,359,360,361]. Although challenging, if successful, this approach would greatly weaken the ability of oncoviral oncoproteins to promote cellular transformation.

With respect to targeting specific host-cell oncogenic drivers induced by oncoviral dysregulation of host-cell splicing, STAT3 is a critical oncogenic driver in EBV-induced cancers and, although there are currently no approved inhibitors, represents an important potential target [362]. SRSF10 is an oncogenic driver in HPV-induced cervical and head and neck squamous cell carcinomas and although there are currently no approved inhibitors, selective inhibitors such as IC8 are promising [46,363]. The alternative TrkAIII splice variant may act as an actionable oncogenic driver in MCPyV-positive Merkel cell carcinomas and could be targeted by approved Trk inhibitors [33,159,364]. Oncoviral activation of the PI3K/Akt pathway by various alternative splicing events may also represent a target for approved PI3K and Akt inhibitors [365,366,367].

### 5.3. Therapeutic Modulation of the Spliceosome

With respect to potential strategies to alter oncoviral-induced aberrant host-cell oncogenic splicing, sspliceosome modulators represent a potential therapeutic strategy based on the direct inhibition or disruption of the spliceosome, which is responsible for pre-mRNA processing. Tumor cells, especially those transformed by oncogenic viruses, often exhibit a splicing addiction and require rapid and precise spliceosome activity to produce viral oncoproteins and pro-survival cellular isoforms. This vulnerability makes tumor cells significantly more sensitive to pharmacological intervention at the spliceosome level than healthy tissue [368,369].

SF3B1 inhibitors include pladienolide B, E7107 and H3B-8800. By binding the SF3B1 subunit of the U2 snRNP, these drugs impair recognition of intronic branch points, leading to widespread splicing defects, intron retention and nonsense-mediated decay of multiple survival genes in cancer cells [368,369]. Given the strong dependence of virus-associated tumors on robust transcription and splicing for the generation of viral oncogenes and tumor-promoting host-cell isoforms, agents that target SF3B1 are promising candidates for viral malignancies [18,369].

The activity of SR proteins, critical for selecting alternative splice sites, is controlled by phosphorylation. Inhibitors of SR protein kinases, such as SRPK1 inhibitors (e.g., SRPIN340), have been shown to remodel VEGF-A splicing by shifting expression away from pro-angiogenic VEGF isoforms towards less angiogenic variants, thereby reducing pathological angiogenesis and Tumor growth in preclinical models [370,371,372].

An emerging application of these modulators is in immunotherapy. Partial inhibition of the spliceosome can induce the production of aberrant or mis-spliced transcripts, leading to the presentation of splicing-derived neoantigens on MHC molecules. These neoepitopes can boost cytotoxic T cell responses and sensitize tumors to immune checkpoint blockade, providing a rationale to combine spliceosome modulators with immunotherapies, including in virus-associated cancers [373,374,375].

### 5.4. Therapeutic Splice-Switching Oligonucleotides

Unlike systemic spliceosome modulators, splice-switching oligonucleotides (SSOs) represent a precision medicine strategy. SSOs are short synthetic nucleic acid sequences (typically 15–30 nucleotides) that bind highly specifically to target sequences on pre-mRNA, such as splice sites (donors or acceptors) or regulatory elements (silencers or enhancers). Their mechanism of action does not involve RNA degradation (unlike siRNAs) but creates a steric hindrance that physically prevents the spliceosome from accessing certain exons, forcing their inclusion or exclusion [226]. In this context, the most relevant applications include: Correction of the angiogenic phenotype: SSOs can, in principle, be designed to modulate VEGF-A splicing by masking specific splice sites or regulatory elements, shifting the balance between pro- and anti-angiogenic isoforms, a strategy that has shown anti-angiogenic effects in preclinical models [262,263]; reprogramming of apoptosis: as previously described, some oncogenic viruses shift the balance of Bcl-2 family genes toward anti-apoptotic variants. SSOs can restore sensitivity to cell death by forcing the splicing of Bcl-xL toward its pro-apoptotic short form Bcl-xS, selectively inducing apoptosis of transformed cells [226,376], and direct targeting of viral oncoproteins: in HPV-induced tumors, antisense or splice-modulating oligonucleotides targeting E6/E7 transcripts could be used to interfere with the production of oncogenic E6 and E7 isoforms, reducing the expression of viral oncoproteins that sustain the malignant phenotype [377,378,379].

Despite the high specificity and therapeutic potential of SSOs, their large-scale clinical application remains hindered by the difficulty of transporting these molecules across biological barriers to the nucleus of target cells. For this reason, research is still strongly focused on the development of efficient delivery systems, such as lipid and peptide conjugates, aimed at improving drug stability and tissue absorption [380].

Although promising, the clinical use of splicing modulators, however, has challenges that include delivery, potential off-target toxicity and narrow therapeutic windows. Solid tumors exhibit high interstitial fluid pressures and inadequate vasculatures, reducing efficient delivery. Splicing modulators, furthermore, may also influence the splicing of normal genes, resulting in toxicity from unintended protein isoforms. High tumor cell adaptability may also bypass modulators by hijacking other splicing factors, or by rapid alterations in DNA methylation and epi-transcriptomics. These problems have prompted research into alternative splicing modulator chemistries and novel delivery systems, with progress reported in the development of small molecules with improved biodistribution and viral vectors that convey antisense sequences [381,382,383,384,385].

## 6. Conclusions

This review highlights the many ways oncogenic viruses hijack, corrupt and exploit host-cell splicing machinery to induce and promote aberrant splicing events that impact all of the hallmarks of cancer. They do this by either interacting directly with or influencing the expression and activity of major host-cell splicing factors, to regulate oncoviral protein production essential for viral replication, maintenance of viral genomes and immune evasion but also remodel host-cell alternatively spliced transcriptomes, generating aberrant isoforms that impact sustained proliferation, inactivate tumor suppressors and tumor suppressor signaling pathways, replicative immortality, tumor-associated angiogenesis, metabolic adaptation, genomic instability, immune evasion and tumor promoting inflammation. The complex molecular mechanisms by which different group 1 carcinogenic viruses influence the host-cell splicing machinery to reprogram host transcriptomes reveals potential splicing-centric vulnerabilities that could be exploited for therapeutic purposes by small molecules, antisense oligonucleotides and/or by CRISPR technology to block the expression of viral oncoproteins that hijack and corrupt the host-cell splicing machinery, to modify corrupted spliceosomes to counteract oncoprotein-induced changes in alternative splicing, and/or to inhibit specific aberrant host-cell tumor-driving oncogenes or oncogenic pathways, activated by oncovirus-induced alternative splicing.

## Figures and Tables

**Table 1:** Similarities and differences between the oncogenic gammaherpesviruses EBV (HHV-4) and KSHV (HHV-8).

	EBV/HHV-4	KSHV/HHV-8
Primary Cell Tropism	B-lymphocytes Epithelial cells	Endothelial cells B-lymphocytes
Associated Malignancies	Burkitt lymphoma Nasopharyngeal carcinoma Hodgkin lymphoma	Kaposi sarcoma Primary effusion lymphoma Multicentric Castleman disease
Key Oncoproteins	EBNA1: Binds viral genome EBNA2: B-cell immortalization LMP1: CD40 mimic/NF-κB LMP2: PI3K/Akt	LANA: Inactivates p53 and Rb v-FLIP: Inhibits apoptosis v-Cyclin: Drives cell cycle (G1 to S phase)

**Table 2:** Summarization of the characteristics of human carcinogenic viruses.

Oncogenic Virus	Genome Type	Viral Family	Associated Human Cancers
Epstein–Barr virus (EBV)	dsDNA	Herpesviridae	Burkitt lymphoma, Hodgkin lymphoma, nasopharyngeal carcinoma, gastric carcinoma.
Human papillomavirus (HPV)	dsDNA	Papillomaviridae	Cervical cancer, anal cancer, oropharyngeal cancer, vaginal, vulvar and penile cancers
Hepatitis B virus (HBV)	Partially dsDNA	Hepadnaviridae	Hepatocellular carcinoma (HCC)
Hepatitis C virus (HCV)	ssRNA	Flaviviridae	Hepatocellular carcinoma (HCC)
Hepatitis D virus (HDV)	ssRNA	Subviral satellite virus	Hepatocellular carcinoma (HCC)
Human T-lymphotropic virus 1 (HTLV-1)	ssRNA	Retroviridae	Adult T-cell leukemia (ATL)
Merkel cell polyomavirus (MCPyV)	dsDNA	Polyomaviridae	Merkel cell carcinoma (MCC)
Kaposi sarcoma-associated herpesvirus (KSHV/HHV-8)	dsDNA	Herpesviridae	Kaposi sarcoma, primary effusion lymphoma, multicentric Castleman disease

Abbreviations: dsDNA, double-stranded DNA; ssRNA, positive-sense single-stranded RNA.

## Data Availability

No new data were created or analyzed in this study.

## Abbreviations

The following abbreviations are used in this manuscript: IARC: International Agency for Research on Cancer; snRNPs: small nuclear ribonucleoproteins; BPS: branch point; PPT: polypyrimidine tract; SR: serine/arginine-rich; EBV: Epstein–Barr virus; HBV: hepatitis B virus; ECV: hepatitis C virus; HDV: hepatitis D virus; KSHV/HHV-8: Kaposi sarcoma-associated herpesvirus; MCPyV: Merkel cell polyomavirus; HTLV-1: human T-lymphotropic virus type-1; HIV: human immunodeficiency virus 1; HPV: human papillomavirus; SV40: Simian virus 40; HR-HPV: high-risk human papillomavirus; p53: tumor protein; pRb: retinoblastoma protein; LMP1: Epstein–Barr virus latent membrane protein 1; MAPK: mitogen-activated protein kinase; PI3K: phosphoinositide 3-kinase; Akt: Ak strain transforming—protein kinase B; STAT: signal transducer and activator of transcription; lyLMP1: truncated lytic Epstein–Barr virus latent membrane protein 1; p21/cip1/waf1: cyclin dependent kinase inhibitor; SRSF10: serine/arginine-rich splicing factor 10; BCLAF1-L: splice variant of BCL2 associated transcription factor 1; SREK1: splicing regulatory glutamic acid and lysine rich protein 1; SREK1L: splice variant of splicing regulatory glutamic acid and lysine rich protein 1; RPL28: ribosomal protein L28; mTOR: mechanistic target of rapamycin; HBx: hepatitis B virus X protein; FAM50A: Family with sequence similarity 50 member A; SHP2: Src homology region 2 domain-containing phosphatase-2; STAT3: signal transducer and activator of transcription 3; NF-YA: nuclear transcription factor Y subunit alpha; TERT: telomerase reverse transcriptase; hTERT: human telomerase reverse transcriptase; ASF/SF2: alternative splicing factor 1 or pre-mRNA-splicing factor; HERV: human endogenous retroviral; HERV-K: endogenous retrovirus group K; Np9: endogenous retrovirus group K member 10 Np9 protein; Rec: endogenous retrovirus group K member 19 Rec protein; HERV-W: human endogenous retrovirus-W; EGF: epidermal growth factor; BZLF1: BamHI Z fragment leftward open reading frame 1; EBNA: Epstein–Barr virus nuclear antigen; LANA: latency-associated nuclear antigen; v-IRF-1: viral interferon regulatory factor 1; v-IRF-3: viral interferon regulatory factor 3; Tax: trans-activating transcriptional regulatory protein of HTLV-1; p300: E1A-associated protein p300; CBP: CREB-binding protein; FBXW7: F-box/WD repeat-containing protein 7; c-Myc: cellular myelocytomatosis oncogene; NS5A: non-structural protein 5A; HIF: hypoxia-inducible factor; VHL: Von Hippel–Lindau tumor suppressor; HDAC6: histone deacetylase 6; H4K20me2: histone H4 lysine 20 dimethylation; H2AK15ub: ubiquitinated histone H2A lysine 15; vBcl-2: viral Bcl-2; BHRF1: apoptosis regulator BHRF1; BALF1: BamHI-A leftward fragment 1; BIM: Bcl-2-interacting mediator of cell death; PUMA: p53 upregulated modulator of apoptosis; BID: BH3-interacting domain death agonist; BAK: Bcl-2 homologous antagonist/killer; IAPs: inhibitor of apoptosis proteins; Bcl-2: B-cell leukemia/lymphoma 2 protein; Bcl-xL: B-cell lymphoma-extra-large; Mcl-1: induced myeloid leukemia cell differentiation protein; IAP2: Cellular inhibitor of apoptosis 2; PERK: protein kinase R-Like endoplasmic reticulum kinase; ATF4: activating transcription factor; ER: endoplasmic reticulum; RBM5: RNA-binding protein 5; cFLIP: cellular FLICE-like inhibitory protein; SF3B155: splicing factor 3B subunit 1; LMP-2A: synaptotagmin-like protein 2; BARF1: BamHI-A rightward frame 1; Mcl-1L: myeloid cell leukemia 1long form; HBZ: HTLV-1 bZIP factor; U2AF: U2 small nuclear RNA auxiliary factor 2; RAD17: cell cycle checkpoint protein RAD17; RAD51: radiation sensitive protein RAD51 recombinase; RIF-1: replication timing regulatory factor 1; BRCA1: breast cancer type 1 susceptibility protein; NF-kB: k-light-chain-enhancer of activated B cells; SRPK1: serine-arginine protein kinase 1; vFLIP: viral FLICE-inhibitory protein; MMP-9: Matrix metalloproteinase-9; Tie-2: Angiopoietin-1 receptor; FGFR2: fibroblast growth factor receptor 2; RON: macrophage stimulating 1 receptor; CEACAM1: carcinoembryonic antigen-related cell adhesion molecule 1; PKM1/M2: pyruvate kinase isozymes; ORF57: open reading frame 57; NADPH: nicotinamide adenine dinucleotide phosphate; AICF: APOBEC1 complementation factor; CTLA4: cytotoxic T-lymphocyte-associated protein 4; CD80: cluster of differentiation 80; CD86: cluster of differentiation 86; PDCD1: programmed cell death 1; PD-L1: programmed death-ligand 1; PD-1: programmed cell death protein 1; PD-L2: programmed cell death 1 ligand 2; CD47: cluster of differentiation 47; Necl-5: nectin-like protein 5; NK: natural killer cells; NUMB: endocytic adaptor protein; ORF45: open reading frame 45; IRF7: interferon regulatory factor 7; TBK1: TANK-binding kinase 1; IKKε: inhibitor of nuclear factor kappa-B kinase subunit epsilon; STING: stimulator of interferon genes; BIN1: bridging integrator 1; HERV-W: human endogenous retrovirus W; WPB11: WW domain-binding protein 11; CEP: centrosomal protein; E2F: E2 promoter-binding factor; BCLAF1: BCL2-associated transcription factor; IL1RAP: interleukin 1 receptor accessory protein; ANXA1: annexin A1; PKM2: pyruvate kinase isozyme M2; EBER: Epstein–Barr virus-encoded small RNA; SRp20: serine/arginine-rich splicing factor 3; RBM4: RNA binding motif protein 4; CBF1: C-promoter binding factor 1; ABCF1: ATP binding cassette subfamily F member 1; HMGA1: high mobility group AT-hook 1; RBM10: RNA binding motif protein 10; RBM14: RNA binding motif protein 14; PABPC4: poly(A) binding protein cytoplasmic 4; MDM2: mouse double minute 2 homolog; DDX3: DEAD-box helicase; BCAT2: branched-chain-amino-acid aminotransferase; ECHDC2: enoyl-CoA hydratase domain-containing protein 2; HLA: human leukocyte antigen; IP6K2: inositol hexakisphosphate kinase 2; ALTO: alternative large tumor open reading frame; IL1beta: interleukin-1 beta; NSR: insulin receptor; IRS2: insulin receptor substrate 2; FOXC2: forkhead box protein C2; PRKCA: protein kinase C alpha; SOCS7: suppressor of cytokine signaling 7; AIDS: acquired immunodeficiency; CCR5: C-C chemokine receptor type 5; CXCR4: C-X-C chemokine receptor type 4; VEGF: vascular endothelial growth factor; SF3b155: splicing factor 3b protein 155; RBM5: RNA binding motif protein 5.
